# Metabolic engineering of *Escherichia coli* for production of n-butanol from crude glycerol

**DOI:** 10.1186/s13068-017-0857-2

**Published:** 2017-07-04

**Authors:** Mukesh Saini, Ze Win Wang, Chung-Jen Chiang, Yun-Peng Chao

**Affiliations:** 1 Department of Chemical Engineering, Feng Chia University, 100 Wenhwa Road, Taichung, 40724 Taiwan; 2 Department of Medical Laboratory Science and Biotechnology, China Medical University, No. 91, Hsueh-Shih Road, Taichung, 40402 Taiwan; 3 Department of Health and Nutrition Biotechnology, Asia University, Taichung, 41354 Taiwan; 4 Department of Medical Research, China Medical University Hospital, Taichung, 40447 Taiwan

**Keywords:** Metabolic engineering, Central metabolism, n-Butanol, Crude glycerol

## Abstract

**Background:**

Crude glycerol in the waste stream of the biodiesel production process is an abundant and renewable resource. However, the glycerol-based industry is usually afflicted by the cost for refinement of crude glycerol. This issue can be addressed by developing a microbial process to convert crude glycerol to value-added chemicals. In this study, *Escherichia coli* was implemented for the production of n-butanol based on the reduced nature of glycerol.

**Results:**

The central metabolism of *E. coli* was rewired to improve the efficiency of glycerol metabolism and provide the reductive need for n-butanol in *E. coli*. This was carried out in several steps by (1) forcing the glycolytic flux through the oxidation pathway of pyruvate, (2) directing the gluconeogenic flux into the oxidative pentose phosphate pathway, (3) enhancing the anaerobic catabolism for glycerol, and (4) moderately suppressing the tricarboxylic acid cycle. Under the microaerobic condition, the engineered strain enabled the production of 6.9 g/L n-butanol from 20 g/L crude glycerol. The conversion yield and the productivity reach 87% of the theoretical yield and 0.18 g/L/h, respectively.

**Conclusions:**

The approach by rational rewiring of metabolic pathways enables *E. coli* to synthesize n-butanol from glycerol in an efficient way. Our proposed strategies illustrate the feasibility of manipulating key metabolic nodes at the junction of the central catabolism. As a result, it renders the intracellular redox state adjustable for various purposes. Overall, the developed technology platform may be useful for the economic viability of the glycerol-related industry.

**Electronic supplementary material:**

The online version of this article (doi:10.1186/s13068-017-0857-2) contains supplementary material, which is available to authorized users.

## Background

Fossil fuels are afflicted by their unsustainability and excessive emission of greenhouse gas after use, which has overshadowed our daily life. It has inevitably urged the requirement for biofuels from renewable resources as crude oil replacement because they are sustainable and environment-friendly [1]. Among alternative fuels, n-butanol is of particular interest while it possesses a property superior to ethanol in terms of energy density, volatility, and hygroscopicity [2]. Moreover, n-butanol can be transported by the existing infrastructure and readily used to fuel vehicle motors after blended with gasoline [3]. *Clostridium* species have long been employed for the mass production of n-butanol, known as the acetone–butanol–ethanol (ABE) fermentation process [4]. This fermentation scheme starts with the acidogenesis followed by the solventogenesis. However, this biphasic production pattern is susceptible to the environmental variation and renders the operation of the ABE fermentation technically challenging.

Many surrogate microbes have been successfully illustrated for the n-butanol production after recruitment of the clostridial CoA-dependent pathway for n-butanol [5–8]. In spite of their feasibility, these research efforts are still discouraged by a low production titer. It is recognized that the redox balance of NADH and NAD^+^ favors the reductive production of n-butanol. The challenge is that the NADH output from glucose catabolism is insufficient to meet the NADH requirement for the synthesis of n-butanol. This issue has been addressed in *Escherichia coli* by enhancement of the pyruvate dehydrogenase (PDH)- and formate dehydrogenase (FDH)-catalyzed reaction steps in glycolysis. As a result, the approach increases the intracellular NADH level and leads to a higher production titer of n-butanol [9–11]. We have tackled the problem by building a redox-balanced synthetic pathway which is distributed into two *E. coli* strains [12]. As recognized, the central metabolism consists of fueling pathways that dictate the availability of NADH. Our alternative strategy has therefore rerouted the central pathways involving glycolysis, the pentose phosphate (PP) pathway, and the tricarboxylic acid (TCA) cycle to modulate the intracellular NADH level [13]. Overall, the *E. coli* strains as engineered in our two approaches enable effective production of n-butanol.

A commercialized bioprocess of n-butanol can be readily realized by a cost-effective feedstock. Biodiesel has been utilized as an alternative fuel for transportation. The annual production yield of biodiesel increases substantially with the pressing need for renewable fuels. Glycerol is a byproduct generated in the production process of biodiesel. The extending market demand has resulted in a large amount of glycerol currently available in the market [14]. The glycerol surplus greatly reduces the price of crude glycerol, which negatively affects the economic viability of the glycerol-producing, oleochemical, and biodiesel industries. The situation is even worsened by the additional cost for disposal of glycerol waste. Therefore, it is appealing to develop a technology platform that converts crude glycerol to the value-added products [15]. Used as a feedstock, glycerol is attractive because it has the highly reduced carbon atoms and enables generation of more reducing equivalents than glucose [16]. This advantage of glycerol makes it more favorable for production of reduced compounds. However, glycerol metabolism is less effective than glucose metabolism in *E. coli*. In this study, *E. coli* was engineered for the production of n-butanol from glycerol. This was systematically carried out by manipulation of central metabolism and glycerol catabolism. Consequently, the proposed approach conferred the strain with the ability to produce n-butanol from crude glycerol in an effective manner.

## Results and discussion

### Microaerobic production of n-butanol from glycerol

**Fig. 1:**
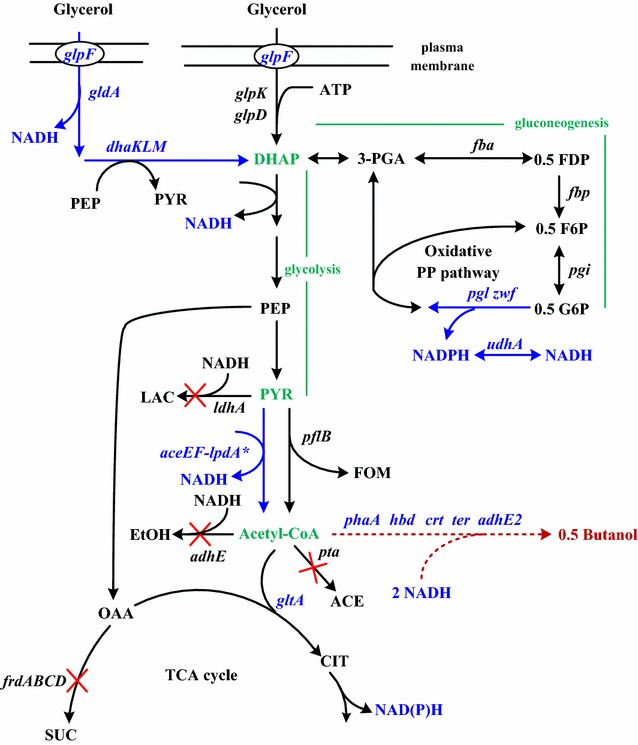
The central metabolic pathways of *E. coli* connecting glycerol catabolism to n-butanol synthesis. The catabolic route of glycerol includes the *glpK*-*glpD* and the *gldA*-*dhaKLM* pathways. The heterologous pathway for the synthesis of n-butanol is composed of *phaA*, *hbd*, *crt*, *ter*, and *adhE2* genes (*dotted line*). The genes involved in the metabolic pathways: *aceEF*-lpdA*: pyruvate dehydrogenase complex; *adhE*, aldehyde–alcohol dehydrogenase; *adhE2*, butyraldehyde–butanol dehydrogenase; *crt*, crotonse; *hbd*, 3-hydroxybutyryl-CoA dehydrogenase; *ldhA*, lactate dehydrogenase; *fba*, fructose bisphosphate aldolase; *fbp*, fructose 1,6-bisphosphatase; *frdABCD*, fumarate reductase; *pflB*, pyruvate-formate lyase; *gltA*, citrate synthase; *glpF*, glycerol facilitator; *gldA*, glycerol dehydrogenase; *dhaKLM*, dihydroxyacetone kinase; *glpK*, glycerol kinase; *glpD*, glycerol 3-phosphate dehydrogenase; *pgi*, phosphoglucose isomerase; *pgl*, lactonase; *phaA*, acetoacetyl-CoA thiolase; *pta*, phosphate acetyltransferase; *ter*, trans-enoyl-CoA reductase; *udhA*, transhydrogenase; *zwf*, glucose-6-phosphate dehydrogenase. The undesired genes in the pathways are deleted as marked with “X.” *ACE* acetate; *EtOH* ethanol; *DHAP* dihydroxyacetone phosphate; *FDP* fructose 1,6-bisphosphate; *F6P* fructose-6-phosphate; *LAC* lactate; *FOM* formate; *G6P* glucose-6-phosphate; *CIT* citrate; *OAA* oxaloacetate; *PEP* phosphoenolpyruvate; *3-PGA* 3-phosphoglyceraldehyde; *PYR* pyruvate; *SUC* succinate

Strain BuT-8 harbors a functional pathway for the synthesis of n-butanol (Table 1) [12]. This heterologous CoA-dependent pathway consists of *hbd*, *crt,* and *adhE2* from *Clostridium acetobutylicum*, *phaA* from *Cupriavidus necator*, and *ter* from *Treponema denticola* (Fig. 1). Moreover, it lacks the endogenous *adhE*, *ldhA*, *pta*, and *frdA* genes responsible for the production of mixed acids. This helps to reduce carbon waste and increase NADH availability. The microaerobic utilization of glycerol in *E. coli* is far superior to the fermentative metabolism [16]. Therefore, the microaerobic production of n-butanol from crude glycerol was investigated in this study. A producer strain was developed starting with strain BuT-8. It is well recognized that the efficient production of n-butanol requires more available NADH [10]. According to the previous study [16], *pflB* plays a main role in the oxidation of pyruvate to acetyl-CoA during the microaerobic utilization of glycerol. In contrast to the PflB counterpart, PDH complex (encoded by *aceEF*-lpdA*) mediates the pyruvate oxidation with concurrent generation of NADH. Therefore, PDH in the strain was enhanced to compete with PflB for more NADH production. In addition, the gluconeogensis involving *fba*, *fbp*, and *pgi* occurs in *E. coli* during the microaerobic growth on glycerol [16]. Accordingly, *zwf* and *pgl* were augmented in the strain to direct the gluconeogenic carbon flux into the PP pathway for generation of NADPH. NADPH was then converted to NADH in the strain equipped with the elevated *udhA* [17]. Finally, the construction gave rise to strain BuT-12A.

**Table 1:** The *E. coli* strains applied in this study

Strain	Characteristic	Source
BuT-8	Δ*frdA* ɸ80*attB*::PλP_L_-*ter* λ*attB*::PλP_L_-*crt* Δ*adhE*::ɸ80*attB*::PλP_L_-*pha*-*hbd* Δ*ldhA*::λ*attB*::PλP_L_-*adhE2*	12
BuT-12A	as BuT-8 ∆*lpdA* λ*attB*::PλP_L_-*lpdA* ^*^ PλP_L_-*aceEF* PλP_L_-*zwf* Δ*atoD*::PλP_L_-*pgl* PλP_L_-*UdhA*	This study
BuT-12-2	as BuT-12A PλP_L_-*gldA* PλP_L_-*dhaKLM*	This study
BuT-12-3	as BuT-12-2 Δ*zwf*	This study
BuT-16	as BuT-12A *lacO*-*gltA*	This study

lpdA* the mutant, *lpdA* exhibiting insensitivity to NADH

**Fig. 2:**
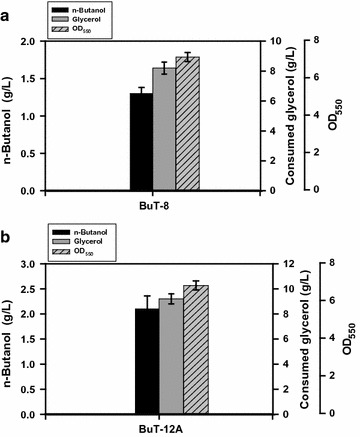
Microaerobic production of n-butanol in strains with the amplification of the fueling pathways. The *E. coli* strains were grown in M9Y medium containing 20 g/L pure glycerol and the fermentations were carried out for 24 h. The experiments were conducted in triplicate. *Keys* **a** the fermentation of strain BuT-8; **b** the fermentation of strain BuT-12A

Pure glycerol was first used for illustration. The microaerobic production of n-butanol was carried out using the shake-flask culture of strain BuT-12A while strain BuT-8 served as a control. As shown in Fig. 2a, b, strain BuT-12A enabled production of 60% more n-butanol than the control strain (2.1 vs. 1.3 g/L) at 24 h of fermentation. The result leads to productivity of 0.09 g/L-h and the conversion yield of 0.23 g/g (Table 2), which accounts for 57.2% of the theoretical yield (ca. 0.40 g/g glycerol).

**Table 2:** Summary of the fermentation kinetics for producer strains

Strain	*P*_B_	*Y*_B/G_	Gene product targeted for manipulation					
			PDH	Zwf	Pgl	GldA	DhaKLM	GltA
BuT-8	0.054	0.16	W	W	W	W	W	W
BuT-12A	0.09	0.23	+	+	+	W	W	W
BuT-12-2	0.13	0.29	+	+	+	+	+	+
BuT-12-3	0.14	0.32	+	−	+	+	+	W
BuT-16	0.18	0.35	+	+	+	+	+	<
	0.24*	0.34*						

The fermentation was carried out with the cell density at OD_550_ of 0.2. Strain BuT-16 was grown in M9Y medium containing crude glycerol of 20 g/L for 40 h. The others were cultured on pure glycerol of 20 g/L for 24 h. The development course of producer strains for the production of n-butanol was shown in Additional file 1: Fig. S1. Note: W, wild type; +, enhancement; −, absence; <, suppression; *P*_B_, n-butanol productivity (g/L/h); *Y*_B/G_, conversion yield of n-butanol on glycerol (g/g)

* The fermentation was conducted with the cell density at OD_550_ of 5 and crude glycerol of 30 g/L for 36 h

## Enhancement of the anaerobic glycerol catabolism

**Fig. 3:**
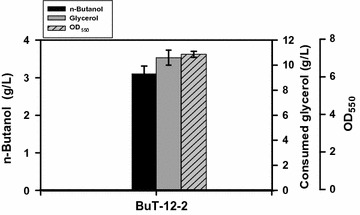
Microaerobic production of n-butanol in the strain with the enhanced glycerol catabolism. *Escherichia coli* strain BuT-12-2 was engineered by enhancement of the *gldA*-*dhaKLM* catabolic route. The strain was grown in M9Y medium containing 20 g/L pure glycerol and the fermentations were carried out for 24 h. The experiments were conducted in triplicate

As indicated in Fig. 1, two catabolic pathways of glycerol existing in *E. coli* include the *glpK*-*glpD* and the *gldA*-*dhaKLM* routes. The former pathway is responsible for the aerobic catabolism of glycerol while the latter route prevails under the anaerobic condition [18]. In addition, the *gldA*-*dhaKLM* pathway that mediates the conversion of glycerol to dihydroxyacetone phosphate (DHAP) generates extra NADH. Direction of more glycerol into the *gldA*-*dhaKLM* pathway is expected to increase the intracellular NADH, which may favor the n-butanol production. Strain BuT-12-2 was thus obtained by genomic fusion of a strong promoter (PλP_L_) with *gldA* and the *dhaKLM* operon to enhance their expression levels in strain BuT-12A. In comparison with strain BuT-12A, strain BuT-12-2 exhibited a 18-fold more GldA activity (0.306 vs. 0.016 U/mg protein) and a 2.5-fold more DhaKLM activity (0.021 vs. 0.006 U/mg protein). The microaerobic culturing of strain BuT-12-2 was conducted in a similar way. At the end of fermentation, the strain utilized 10.5 g/L glycerol and produced 3 g/L n-butanol at 24 h (Fig. 3). The conversion yield (ca. 0.29 g/g) and productivity (ca. 0.13 g/L/h) for strain BuT-12-2 are increased by 26 and 44% than those for BuT-12A, respectively. For microaerobic utilization of glycerol in *E. coli*, the *glpK*-*glpD* pathway is absolutely required while the glycerol utilization rate can be reduced by 50% in the absence of the *gldA*-*dhaKLM* route [16]. In the current case, the significance of the *gldA*-*dhaKLM* pathway is acknowledged by its positive contribution to the microaerobic production of n-butanol from glycerol.

## Restriction of the TCA cycle

**Fig. 4:**
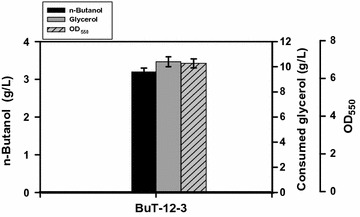
Microaerobic production of n-butanol in the strain with the gluconeogenic flux via the reductive PP pathway. *Escherichia coli* strain BuT-12-3 was engineered by the removal of *zwf*. The strain was grown in M9Y medium containing 20 g/L pure glycerol and the fermentations were carried out for 24 h. The experiments were conducted in triplicate

Strain BuT-12-2 is engineered with redistribution of carbon flux in central metabolism interconnecting the anaerobic catabolism of glycerol, the oxidative PP pathway, and the oxidative pathway of pyruvate. As revealed in Fig. 1, the synthesis for one molecule of n-butanol requires 4 molecules of NADH and 2 molecules of acetyl-CoA. The *gldA*-*dhaKLM* pathway of glycerol catabolism produces 2 molecules of pyruvate and 4 mol of NADH at the expense of 2 molecules of glycerol, which suffices the need of NADH. Extra NADH is generated after a part of carbon flux is driven into the PDH-based route and the oxidative PP pathway. Therefore, strain BuT-12-2 likely possesses a surplus of NADH. Note that the catabolic flux of glycerol bifurcates at the DHAP node where the gluconeogenic and the glycolytic flux move towards the PP pathway and the TCA cycle (Fig. 1), respectively. To address the issue, the PP pathway was first targeted to engineer. In addition to provision of precursors for nucleic acids and aromatic amino acids, the PP pathway generates NADPH for use in the reductive biosynthesis. The PP pathway undergoes the oxidative and nonoxidative routes in response to the intracellular requirement for NADPH [19]. Strain BuT-12-2 with enhanced *zwf*-*pgl* mainly assumes the oxidative PP pathway with generation of NADPH which is converted to NADH mediated by UdhA. To change the operation mode in the PP pathway, *zwf* was deleted in strain BuT-12-2 to obtain strain BuT-12-3. Consequently, the gluconeogenic carbon flux in strain BuT-12-3 is forced through the nonoxidative PP pathway without generation of the reducing equivalent. Strain BuT-12-3 was grown under the microaerobic condition and finally produced 3.4 g/L n-butanol at 24 h (Fig. 4). The result leads to a marginal improvement in the conversion yield and productivity (Table 2). It indicates that the oxidative PP pathway is less significant to provide reducing equivalents for the n-butanol synthesis.

**Fig. 5:**
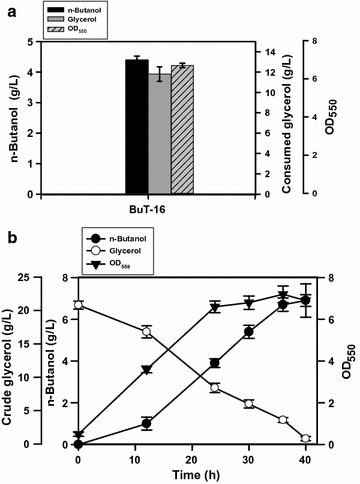
Microaerobic production of n-butanol in the strain with the suppressed TCA cycle. *Escherichia coli* strain BuT-16 was engineered by suppression of the *gltA* gene. The strain was grown in M9Y medium containing either 20 g/L pure glycerol or crude glycerol. The fermentations using pure glycerol and crude glycerol were carried out for 24 and 40 h, respectively. The experiments were conducted in triplicate. *Keys* **a** the fermentation with pure glycerol; **b** the fermentation with crude glycerol

Next, the TCA cycle was manipulated. The TCA cycle operates in an oxygen-responsive way such that the production level of reducing equivalents varies with either the oxidative or the reductive pathway [19]. To restrict the entry of the carbon flux into the TCA cycle, it is useful to conserve acetyl-CoA (the precursor of n-butanol) and reduce the production of reducing equivalents. According to the previous method [13], the *gltA* (encoding citrate synthase) cognate promoter P2 was replaced by *lacO* in strain BuT-12-2 to obtain strain BuT-16. The resulting strain exhibited a 30% lower GltA activity as a result of endogenous LacI-mediated repression. Strain BuT-16 was then cultured and examined for its fermentative performance. Consequently, n-butanol of 4.3 g/L was obtained from 11.5 g/L glycerol at 24 h (Fig. 5a). As compared to strain BuT-12-2, strain BuT-16 displays an increase of around 30 and 40% in the conversion yield and productivity, respectively. Carrying a reduced GltA activity, the glycerol-grown strain BuT-16 grew normally. This is in agreement with the previous study reporting that the growth of *E. coli* on glucose remains unaffected by a 90% decrease in the GltA activity [20].

At last, the performance of strain BuT-16 using crude glycerol was investigated and the fermentation process was prolonged to obtain the maximum production. The strain consumed all crude glycerol (20 g/L) and produced 6.9 g/L n-butanol at 40 h (Fig. 5b), accounting for the conversion yield of 0.35 g/g and productivity of 0.18 g/L/h. These results are comparable to those on glucose (ca. 0.31 g/g and 0.21 g/L/h) as reported [13]. Consequently, strain BuT-16 greatly reduces the production of byproducts and achieves 5-fold more productivity in comparison with its parent strain BuT-8 (Table 3).

**Table 3:** Carbon recovery of fermentation products for engineered strains grown on crude glycerol

Strain	Succinate	Ethanol	Lactate	Acetate	Butyrate	Butanol	Total (%)
BuT-8	5.0	9.9	2.3	4.6	10.4	27.3	59.5
BuT-16	0.8	4.2	0.6	2.2	4.5	57.7	70.0

Carbon recovery was calculated as the molar percent of carbon in products per carbon in consumed glycerol. The data for strain BuT-8 were taken at 72 h of fermentation

## Production of n-butanol with concentrated cell

**Fig. 6:**
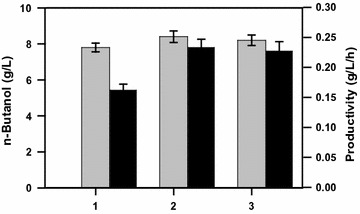
Production of n-butanol with the high cell density. The fermentation was carried out using *E. coli* strain BuT-16 at various cell densities in M9Y medium containing 30 g/L crude glycerol. The maximum production of n-butanol was obtained for the case with OD_550_ of 1 at 48 h and with OD_550_ of 5 or 10 at 36 h. The fermentation of the strain became sluggish after the peak production. The experiments were duplicated. Keys: *1* OD_550_ of 1; *2* OD550 of 5; *3* OD_550_ of 10. *Symbols* production (*gray*); productivity (*black*)

The improvement of the n-butanol production was attempted first by using strain BuT-16 (OD_550_ of 0.2) fed with higher crude glycerol (30 g/L). The strain exhibited a slow consumption of crude glycerol with the productivity reducing to 0.13 g/L/h. This is likely due to the inhibitory effect of the impurity in crude glycerol on the cell [21, 22]. To tackle this problem, the fermentation was carried out using the concentrated cell. As a result, the maximum production titer (8.4 g/L) was obtained with the cell density reaching OD_550_ of 5 at 36 h (Fig. 6). The result leads to the conversion yield of 0.34 g/g and productivity of 0.24 g/L/h. In addition, the approach with a higher cell density (at OD_550_ of 10) was implemented for the production of n-butanol. An alternative one was performed with the cell at OD_550_ of 5 for first 24 h and extra cell was then added to the culture broth (finally reaching OD_550_ at 10). Consequently, these two approaches exhibited similar fermentation performance and neither of them could further improve the maximum production titer and productivity (Fig. 6). The tolerance threshold of n-butanol for *E. coli* is strain-dependent and around 8-10 g/L as reported [23, 24]. Therefore, the strain is likely subject to the toxic effect of n-butanol which could disrupt the cell membrane function and abolish the cell metabolism for continuous fermentation. Obviously, such a toxicity-induced inhibitory effect on the cell cannot be circumvented by using the concentrated cell and nutrient supplement [25].

The implementation of enhanced FDH and PDH remains a common strategy to increase intracellular NADH availability for the n-butanol production in glucose-grown *E. coli*. The results are encouraging in terms of conversion yield and productivity [9–11]. Nevertheless, the super-rich TB medium (mainly 12 g/L tryptone and 24 g/L yeast extract) was employed in these studies and components other than glucose in the medium contributed to 15% of the n-butanol production, which complicates the interpretation of the results [10]. In contrast, n-butanol was merely synthesized from glucose in the M9Y medium as recently illustrated in our studies [12, 13]. With M9Y medium plus glycerol, n-butanol was effectively produced in *E. coli* by rational rewiring of the central metabolism. This involves the approach to force glycerol catabolism via the *gldA*-*dhaKLM* pathway, to channel the glycolytic flux into the pyruvate oxidation route and away from the TCA cycle, and to direct the gluconeogenic flux into the oxidative PP pathway. Consequently, it gives the conversion yield reaching 87% of the theoretical one, the highest value ever reported so far. There are only few studies devoted to the production of n-butanol by genetically modified *E. coli* strains on TB medium plus pure glycerol [7, 26]. One reported the n-butanol production of 0.55 g/L and the productivity of 22.9 mg/L/h by a producer strain deprived of *adhE*, *ldhA*, *frdBC*, and *fnr* [7]. The other one reported the n-butanol production of 0.15 g/L and the productivity of 3.2 mg/L/h by a strain which was equipped with FDH and deficient in *adhE*, *ldhA*, and *frdBC* [26]. Nevertheless, the strategy by either evolving our producer strain to tolerate a high concentration of crude glycerol and n-butanol or implementing the production scheme integrated with the in situ removal of n-butanol technology should provide a promising way to further improve the production titer and productivity.

## Conclusions

The intracellular redox state in microbes is manifested by the interplay of the carbon flux distributed in the central metabolism. In this study, the catabolic flux of glycerol was modulated by manipulating the fueling pathways in the central metabolism. n-Butanol was highly synthesized as a result of the flux redistribution. It suggests that the DHAP, pyruvate, and acetyl-CoA nodes at the junction of the central metabolic pathways play a vital role in the glycerol-based synthesis of n-butanol. In conclusion, our current study and others propose an appealing way to produce a value-added chemical from crude glycerol [27]. Continued efforts towards the advance of the technology platform may provide a solution to the economic viability of the glycerol-related industry.

## Methods

### Bacterial culturing

The microaerobic culturing of bacteria essentially followed the previous report [13]. The overnight cultures were grown on Luria–Bertani medium [28] with 2 g/L glycerol. The cell density was determined with a spectrophotometer set at 550 nm (OD_550_). The seeding cells were inoculated into capped Erlenmeyer flasks (125 mL) containing 50 mL M9Y medium (6 g/L Na_2_HPO_4_, 3 g/L KH_2_PO_4_, 0.5 g/L NaCl, 1 g/L NH_4_Cl, 1 mM MgSO_4_, 0.1 mM CaCl_2,_ and 5 g/L yeast extract) with 20 g/L glycerol. Unless otherwise stated, the initial cell density at OD_550_ of 0.2 was used to start the shake-flask cultures. The bacterial cultures were maintained in an incubator operated at 100 rpm. Crude glycerol was kindly provided by Yeow Hwa Co., Ltd. (Taichung, Taiwan) with the composition of 67% (w/w) glycerol.

### Strain construction

The strains employed in this study are summarized in Table 1. The work for strain construction was carried out with *E. coli* strain BuT-8 [12]. This strain harbors a genomic copy of the n-butanol synthetic pathway under control of the λP_L_ promoter (PλP_L_). To endow the strain with enhanced PDH, *lpdA* of strain BuT-8 was knocked out following the previous report [13]. The PCR DNA containing the partial *lpdA* inserted with the LE*-*kan*-RE* cassette was amplified from plasmid pBlue-Ac-lpdA with primers RC12288-RC12290 [13]. By the act of λ Red-mediated recombination, the PCR DNA was directionally integrated and the associated kanamycin-resistant marker (*kan*) was later removed from the strain’s genome [29]. Furthermore, the NADH-insensitive mutant *lpdA* (lpdA*) consisting of the E354K mutation was integrated into the strain at the λ prophage attachment site. This was conducted with plasmid pLam-LpdA* carrying PλP_L_-lpdA* [13]. Lastly, PλP_L_ was fused with the *aceEF* operon using the PCR DNA amplified from plasmid pPR-aceE with RC12060-RC12086 [13].

In addition, the PCR DNAs were synthesized from plasmids pPR-zwf and pPR-udhA with primers RC11417-RC11418 and RC11419-RC11420 [13], respectively. These DNAs were applied for fusion of *zwf* and *udhA* with PλP_L_ to enhance their expression levels in the PP pathway. Meanwhile, *pgl* was introduced into the strain using plasmid pLoxKm-PL with primers RC13001-RC13293 [13]. To delete *zwf*, the DNA fragment containing the FRT site-flanked *kan* (FRT-*kan*-FRT) cassette was amplified from strain JW1841 (△*zwf*-777::*kan*) using primers RC11404 (cagcagagctcgaatggatcgcgttatc) and RC15108 (gtcagagcaggatgattcac). The DNA cassette was then applied to knock out *zwf* following the reported protocol [29].

Finally, *gldA* and *dhaKLM* were fused with PλP_L_ by introduction of PCR DNAs with two homologous extensions into the strain. The PCR DNAs were obtained from plasmid pPL-Gn with primers Gld1-Gld2 and Dha1-Dha2 [29]. Furthermore, the P2 promoter of *gltA* was replaced with *lacO*. This was carried out by electroporation of the PCR DNA which was amplified from plasmid pB-gltO-Cm with primers RC13197-RC13201 [13].

### Analytical methods

Based on the previous methods [12, 22], glycerol was analyzed using High-Performance Liquid Chromatography equipped with Reflective Index RID-10A (Shimadzu, Japan) while n-butanol was measured by Gas Chromatograph Trace 1300 (Thermo Scientific, USA). Cell-free extract (CFX) was obtained by disrupting bacterial cultures with sonication to recover the supernatant after centrifugation. The total protein content in CFX was then analyzed by using Bio-Rad protein assay kit. The GldA activity was determined by monitoring the reduction of NAD^+^ at 340 nm at room temperature. The reaction solution (1 mL) contains 100 mM potassium carbonate buffer (pH 7.9), 100 mM glycerol, 33 mM ammonium sulfate, and 1 mM NAD^+^. The reaction was initiated by adding 100 µL CFX to the reaction solution. Similar to the GldA activity assay, the DhaKLM activity was measured following the reduction of NAD^+^. The reaction solution (1 mL) consists of 100 mM potassium carbonate buffer (pH 9), 100 mM glycerol, 1 mM MgCl_2_, 2 mM dithiothreitol, 1 mM PEP, and 1 mM NAD^+^. The unit (U) of the enzyme activity was defined as μmole/min. The yield of n-butanol on glycerol was calculated as the production amount of n-butanol (g/L) divided by the consumed amount of glycerol (g/L).

## Additional file

**Additional file 1: Figure S1.** The development course of *E. coli* strains for the microaerobic production of n-butanol based on glycerol.

## Abbreviations

PDH: pyruvate dehydrogenase
FDH: formate dehydrogenase
PP: pentose phosphate
TCA: tricarboxylic acid
DHAP: dihydroxyacetone phosphate
PλP_L_: λP_L_ promoter
CFX: cell-free extract
ACE: acetate
EtOH: ethanol
FDP: fructose 1,6-bisphosphate
F6P: fructose-6-phosphate
LAC: lactate
FOM: formate
G6P: glucose-6-phosphate
CIT: citrate
OAA: oxaloacetate
PEP: phosphoenolpyruvate
3-PGA: 3-phosphoglyceraldehyde
PYR: pyruvate
SUC: succinate
