# Supplementary material for: Metabolic engineering of Escherichia coli for production of n-butanol from crude glycerol
Source: Biotechnol Biofuels. 2017 Jul 4;10:173. doi: 10.1186/s13068-017-0857-2 (PMC5496137; doi:10.1186/s13068-017-0857-2)
Supplement: Supplementary file 1 — Additional file 1: Figure S1. The development course of E. coli strains for the microaerobic production of n-butanol based on glycerol. [file 13068_2017_857_MOESM1_ESM.doc]

**Supplementary Material**

**for**

**Metabolic engineering of *Escherichia coli* for production of n-butanol from crude glycerol**

Mukesh Saini,^1^ Ze Win Wang,^1^ Chung-Jen Chiang,^2*^ Yun-Peng Chao,^1,3,4*^

^1^Department of Chemical Engineering, Feng Chia University

100 Wenhwa Road, Taichung 40724, Taiwan

^2^Department of Medical Laboratory Science and Biotechnology, China Medical University, No. 91, Hsueh-Shih Road, Taichung 40402, Taiwan

^3^Department of Health and Nutrition Biotechnology, Asia University, Taichung 41354, Taiwan

^4^Department of Medical Research, China Medical University Hospital, Taichung 40447, Taiwan

Fig. S1. The development course of *E. coli* strains for the microaerobic production of n-butanol based on glycerol. The data were taken from Figs. 2A, 2B, 4, and 5A.
